# Integrating lipid metabolite analysis with MRI-based transformer and radiomics for early and late stage prediction of oral squamous cell carcinoma

**DOI:** 10.1186/s12885-024-12533-x

**Published:** 2024-07-03

**Authors:** Wen Li, Yang Li, Shiyu Gao, Nengwen Huang, Ikuho Kojima, Taro Kusama, Yanjing Ou, Masahiro Iikubo, Xuegang Niu

**Affiliations:** 1 Department of Oral and Maxillofacial Surgery, The First Affiliated Hospital of Fujian Medical University, Fuzhou, China; 2 School and Hospital of Stomatology, Fujian Medical University, Fuzhou, China; 3 Department of Oral Diagnosis, Tohoku University Graduate School of Dentistry, Sendai, Japan; 4 School of Mathematics and Statistics, Huazhong University of Science and Technology, Wuhan, China; 5 Department of Neurosurgey, National Regional Medical Center, Binhai Campus of the First Affiliated Hospital, Fujian Medical University, Fuzhou, China; 6 Department of Neurosurgey, The First Affiliated Hospital of Fujian Medical University, Fuzhou, China

**Keywords:** Oral squamous cell carcinoma, Radiomics, Vision Transformer, Lipid metabolism

## Abstract

**Background:**

Oral Squamous Cell Carcinoma (OSCC) presents significant diagnostic challenges in its early and late stages. This study aims to utilize preoperative MRI and biochemical indicators of OSCC patients to predict the stage of tumors.

**Methods:**

This study involved 198 patients from two medical centers. A detailed analysis of contrast-enhanced T1-weighted (ceT1W) and T2-weighted (T2W) MRI were conducted, integrating these with biochemical indicators for a comprehensive evaluation. Initially, 42 clinical biochemical indicators were selected for consideration. Through univariate analysis and multivariate analysis, only those indicators with *p*-values less than 0.05 were retained for model development. To extract imaging features, machine learning algorithms in conjunction with Vision Transformer (ViT) techniques were utilized. These features were integrated with biochemical indicators for predictive modeling. The performance of model was evaluated using the Receiver Operating Characteristic (ROC) curve.

**Results:**

After rigorously screening biochemical indicators, four key markers were selected for the model: cholesterol, triglyceride, very low-density lipoprotein cholesterol and chloride. The model, developed using radiomics and deep learning for feature extraction from ceT1W and T2W images, showed a lower Area Under the Curve (AUC) of 0.85 in the validation cohort when using these imaging modalities alone. However, integrating these biochemical indicators improved the model’s performance, increasing the validation cohort AUC to 0.87.

**Conclusion:**

In this study, the performance of the model significantly improved following multimodal fusion, outperforming the single-modality approach.

**Clinical relevance statement:**

This integration of radiomics, ViT models, and lipid metabolite analysis, presents a promising non-invasive technique for predicting the staging of OSCC.

**Supplementary Information:**

The online version contains supplementary material available at 10.1186/s12885-024-12533-x.

## Introduction

Oral Squamous Cell Carcinoma (OSCC) is a major global health challenge, ranking as one of the top ten most common cancers worldwide [1]. This malignancy is known for its aggressive nature and propensity for early lymphatic spread [2, 3]. Early stage OSCC typically presents with a favorable prognosis, with early surgical intervention being the common treatment approach, offering a higher likelihood of successful outcomes. Studies indicate that patients diagnosed with early stage OSCC have an 80% chance of surviving beyond five years [4]. In contrast, late stage OSCC often poses more challenging scenarios, necessitating more aggressive treatment strategies such as a combination of surgery, radiotherapy, chemotherapy, and immunotherapy, with a lower post-treatment survival rate. Therefore, determining whether a patient’s tumor is in an early or late stage at the time of presentation is crucial for physicians in devising appropriate treatment plans.

In management of OSCC, physicians primarily rely on Magnetic Resonance Imaging (MRI) and readily available biochemical indicators, which are the most accessible sources of patient information. In early stages of OSCC, MRI provides detailed assessment of tumor size and depth, which is crucial for surgical planning [5]. As tumor advances, MRI helps evaluate tumor invasiveness, lymph node involvement, and possible metastasis, thereby informing treatment strategies [6]. However, effective interpretation of MRI requires physicians to possess extensive experience in reading scans to discern changes within or surrounding the tumor and lymph nodes. The ability of physicians to visually identify the stage of a tumor-early or late-demands technical sensitivity. Beyond the reliance on imaging modalities, it is imperative for physicians to consider the implications of alterations in specific clinical biochemical indicators. Routinely included in blood tests, these indicators offer critical insights into the body’s inflammatory response and metabolic shifts, serving as potential harbingers of tumor presence and progression [7, 8]. At the same time, changes in the body’s immunity, metabolism, and endocrine also affect the occurrence and development of tumors. Consequently, they often present with a variety of biochemical indicator changes. However, the specificity of these indicators is influenced by numerous other factors, placing high demands on clinical physicians when using them as adjunct diagnostic measures.

The machine learning and deep learning algorithms, harnessing the power of large datasets, are increasingly being applied to enhance the accuracy and efficiency of tumor diagnostic processes [9]. Khanfari et al.’s study showed that using radiomics and deep features from multiparametric MRI can be used to evaluate grade status cancer [10]. In addition to disease diagnosis, generative adversarial networks (GANs) in deep learning can facilitate the conversion between T2 weighted (T2W) fluid attenuation inversion recovery (FLAIR) and T2W MRI images, helping clinical decision-making when alternative sequences or rescanning are not feasible [11]. A particularly promising development in this field is the application of Vision Transformer (ViT) models. Originally designed for tasks in natural image processing, ViTs have shown remarkable success in medical imaging [12]. These models, which use self-attention mechanisms, are adept at handling the complexity and variability inherent in medical images [13]. By learning contextual relationships within the data, ViTs can provide nuanced insights that are critical for the early detection and staging of diseases [14]. In the application of deep learning and machine learning, employing data from other hospitals as an external validation cohort can effectively verify the robustness and generalizability of the model.

This research employs contrast-enhanced T1-weighted (ceT1W) and T2W MRI from patients with OSCC as the primary data source. By applying machine learning and ViT techniques, it effectively extracts key imaging features. These features, when combined with clinical biochemical indicators, form the basis of a logistic regression model designed to accurately predict OSCC at both early and late stages. This study exemplifies the advancement in patient-centered oncology care, highlighting the use of non-invasive, yet highly informative, diagnostic techniques.

## Materials and methods

### Patients

**Fig. 1:**
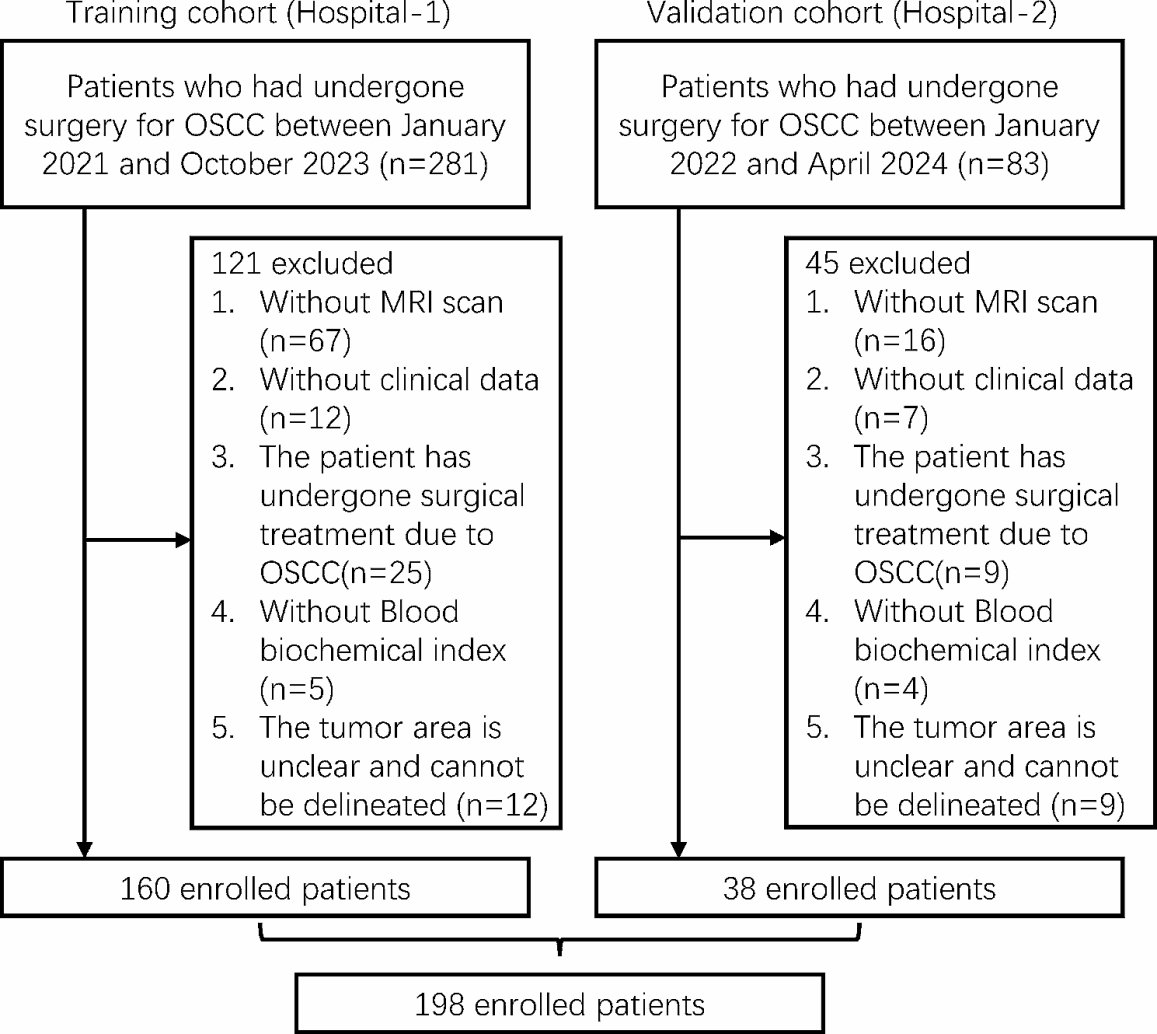
Flow diagram of the study population. OSCC: Oral Squamous Cell Carcinoma; MRI: Magnetic Resonance Imaging

The participant recruitment and study procedures for this retrospective analysis adhered strictly to the ethical guidelines outlined in the 1964 Helsinki Declaration. Ethical clearance was granted by the Ethics Committee (approval number: 2023 − 598). This study incorporated 160 patients diagnosed with OSCC at the First Affiliated Hospital of Fujian Medical University from January 2021 to October 2023, serving as the training cohort. Additionally, 38 patients diagnosed with OSCC at National Regional Medical Center of Binhai Campus of the First Affiliated Hospital, Fujian Medical University (Huashan Hospital Fujian Campus, Fudan University), between January 2022 and April 2024 were included as the validation cohort (Fig. 1). The clinical demographics of the patients, encompassing age, gender, and body mass index (BMI), were methodically documented. Tumor staging was assessed according to the 8th Edition of the Union for International Cancer Control (UICC) Tumor, Node, and Metastasis (TNM) Staging System Manual. The interpretation of TNM staging is based on postoperative pathological reports by two pathologists with more than five years of professional experience and two oral and maxillofacial surgeons with more than five years of clinical experience. Patients were categorized based on their TNM staging: those within stages I-II were classified as early stage, whereas individuals presenting with stages III-IV were designated as late stage patients.

### Analysis of inflammation indices and biochemical indicators

Preoperative assessment involves the measurement of Platelet-to-Lymphocyte Ratio (PLR), Neutrophil-to-Lymphocyte Ratio (NLR), Lymphocyte-to-Monocyte Ratio (LMR), and Systemic Immune-Inflammation Index (SIRI). Concurrently, preoperative evaluation of biochemical indicators in the patient’s blood is performed.

The biochemical tests encompass a comprehensive array of indicators, including: Total Bilirubin (TBIL), Direct Bilirubin (DBIL), Indirect Bilirubin (IBIL), Total Protein (TP), Albumin (ALB), Globulin (GLOB), Albumin/Globulin Ratio (A/G), Alanine Aminotransferase (ALT), Aspartate Aminotransferase (AST), ALT/AST Ratio, Gamma-Glutamyl Transferase (GGT), Lactate Dehydrogenase (LDH), Alkaline Phosphatase (ALP), Creatine Kinase (CK), Creatine Kinase MB Isoenzyme (CK-MB), Urea, Creatinine (CREA), Urea/Creatinine Ratio (URE/CREA), Uric Acid (UA), Glucose (GLU), Cholesterol (CHOL), Triglyceride (TG), High-Density Lipoprotein Cholesterol (HDL-C), High-Density Lipoprotein/Total Cholesterol Ratio (HDL-TC), Low-Density Lipoprotein Cholesterol (LDL-C), Very Low-Density Lipoprotein Cholesterol (VLDL-C), Apolipoprotein A1 (APOA1), Apolipoprotein B (APOB), APOA1/B Ratio, Calcium (Ca), Inorganic Phosphorus (IP), Magnesium (Mg), Bicarbonate (HCO3), Potassium (K), Sodium (Na), Chloride (Cl), Anion Gap (AG), and Glomerular Filtration Rate (GFR).

### Image acquisition and processing

All participants underwent MRI using a 3T superconducting magnetic resonance scanner (Siemens, Germany) equipped with a head and neck array coil. The ceTIW and T2W MRI sequences acquired in this study both incorporate fat suppression techniques. The MRI acquisition parameters were as follows: for T2W images, the repetition time (TR) and echo time (TE) were set at 4000 ms and 79 ms, respectively, with a field of view (FOV) of 220 × 220 mm and a slice thickness of 4 mm. For ceT1W images, the parameters were TR/TE of 400 ms/2.4 ms, an identical FOV of 220 × 220 mm, and a slice thickness of 4 mm.

### Regions of interest (ROI) segmentation and mask dilation

**Fig. 2:**
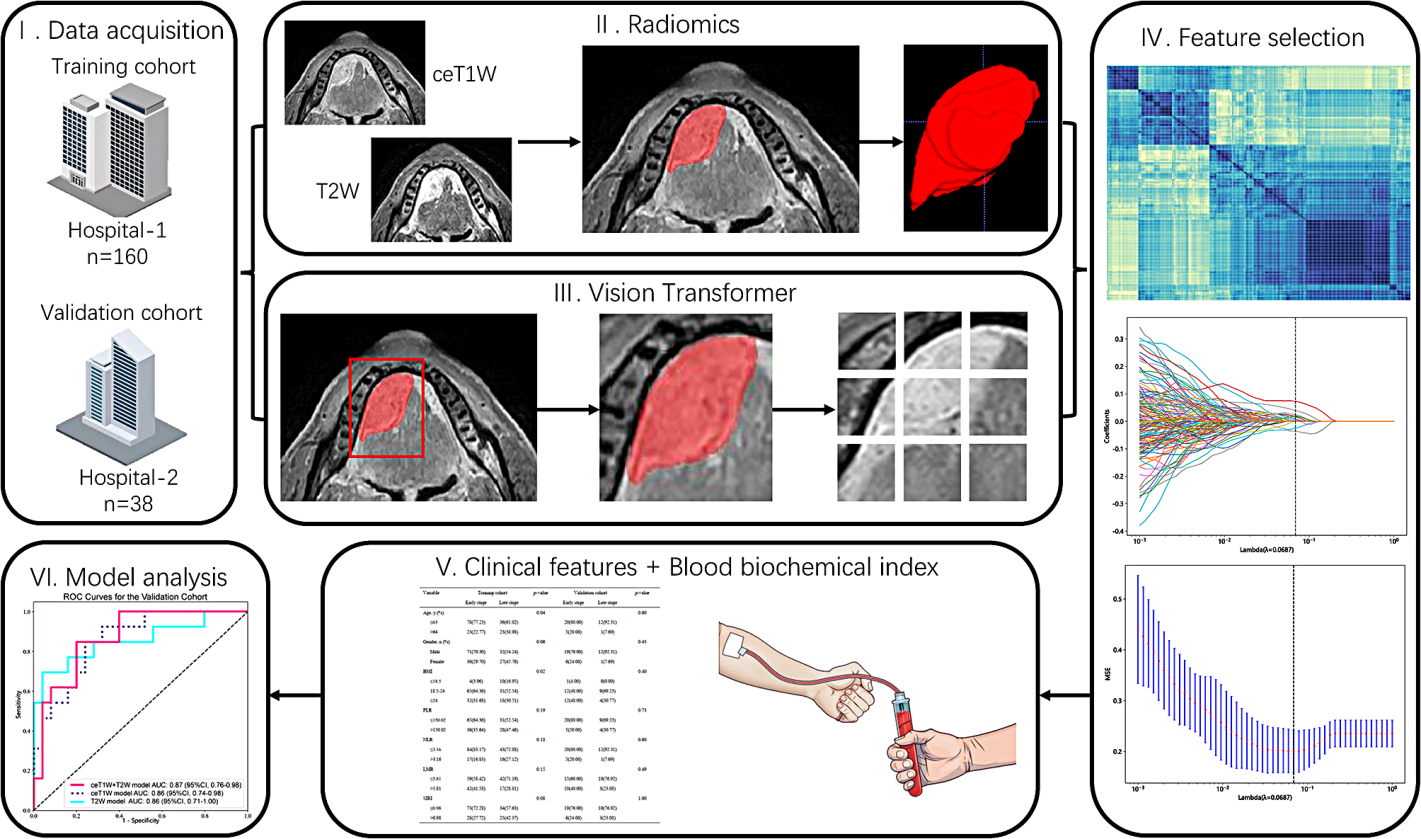
Workflow of the study

Figure 2 illustrates the model construction process. The initial step involved applying N4 bias field correction to MRI scans [15], resizing voxels to 1 mm × 1 mm × 1 mm, and standardizing the images [16]. Lesion-targeted ROIs were delineated slice-by-slice in these scans using ITK-SNAP software (version 3.8.0, www.ITK-SNAP.org). This task was performed by an experienced oral and maxillofacial surgeon, who was blinded to the patients’ clinical data, and the results were verified by a senior oral and maxillofacial surgeon. Using Pyradiomics (version 2.2.0, http://pyradiomics.readthedocs.io) [17], quantitative radiomic features were extracted from the MRI. In total, the study extracted 1,666 radiomics features, comprising 833 features from T2W images and an equal number from ceT1W images. We calculated intraclass correlation coefficients (ICCs) to evaluate the feature extraction consistency by the two specialists. Features showing intra- or inter-observer ICCs under 0.75 were omitted, considering their relative lack of robustness [18].

### Radiomics and deep learning feature extraction

The machine learning features hand-crafted for analysis are broadly divided into three categories: (1) Geometric Features, (2) Intensity Features, and (3) Texture Features. These extracted features include a range of first-order features, as well as several matrix-based features: Gray-Level Co-occurrence Matrix (GLCM), Gray-Level Dependence Matrix (GLDM), Gray-Level Run Length Matrix (GLRLM), Gray-Level Size Zone Matrix (GLSZM), and Neighboring Gray Tone Difference Matrix (NGTDM) features. Additionally, shape features were also considered. For detailed methods on radiomics, please refer to Supplementary Methods.

**Fig. 3:**
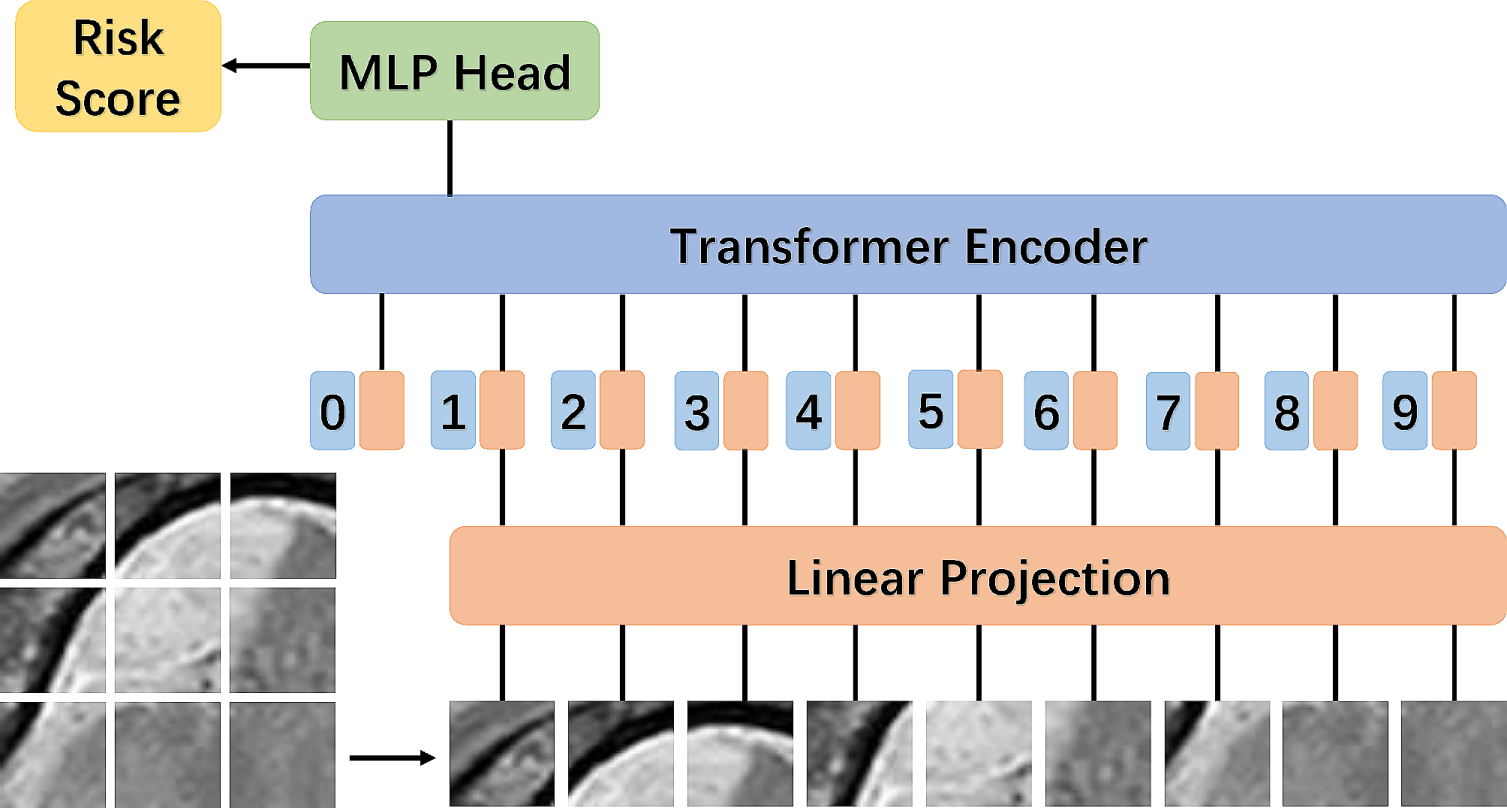
Diagram shows the deep learning model structure. MLP: multilayer perceptron

Deep learning model was trained to predict patient risk scores using segmented tumor volumes derived from preprocessed ceT1W and T2W MRI. The segmentation focused on the ROI specifically centering on the largest cross-section of the tumor, along with an additional 10-pixel margin encompassing the outer edge of the tumor (Fig. 3). An adaptive moment estimation optimizer was implemented with a learning rate of 0.1 for 30 epochs using a batch size of 32. For detailed methods on deep learning, please refer to Supplementary Methods. For this purpose, we utilized a pretrained deep learning (DenseNet121, GoogLeNet, ResNet18, ResNet34 and ViT) models, which had been initially trained on the ImageNet-21 K dataset.

### Feature selection

Prior to detailed analysis, all extracted radiomics and deep learning features underwent standardization to a normal distribution, achieved through z-score normalization. For features exhibiting a normal distribution, Student’s t-tests were applied, with a threshold set to include only those features demonstrating a *p*-value less than 0.05 for subsequent analyses. Spearman’s rank correlation coefficient was employed to ascertain the correlation among features with notable repeatability [19]. In efforts to reduce redundancy, we opted to retain a single feature from each pair that presented a correlation coefficient exceeding 0.9 [20]. Furthermore, a strategic approach of greedy recursive deletion was adopted for feature filtering, aimed at enhancing the informative value of the selected features.

The development of a predictive signature from the discovery dataset employed the Least Absolute Shrinkage and Selection Operator (LASSO) regression model, renowned for its ability to compress regression coefficients towards zero, thereby often reducing coefficients of uncorrelated features to zero. The selection of the optimal regularization parameter, λ, involved using a criterion based on minimization in combination with 10-fold cross-validation. Features with non-zero coefficients, as determined by the LASSO model, were incorporated to construct the regression model and subsequently to formulate the radiomics and deep learning signature. For detailed methods on feature selection results, please refer to Supplementary Methods. The Python *scikit-learn* package [21] was the tool of choice for conducting the LASSO regression modeling.

### Prediction model development

In the analysis of clinical and blood test result variables, univariate logistic regression was employed to assess their association with the differentiation of early and late stage tumors. Variables demonstrating a statistically significant correlation (*p*-value < 0.05) were then incorporated into a multivariate logistic regression analysis. Subsequently, three distinct models were developed: (1) the ceT1W-clinical model, integrating ceT1W radiomics and deep learning features with clinical data, (2) the T2W-clinical model, combining T2W radiomics and deep learning features with clinical data, and (3) the combined model, which encompassed both ceT1W and T2W radiomics, deep learning features, and clinical data. Each of these models was constructed using logistic regression to create a predictive framework.

### Statistical analysis

For data adhering to a normal distribution, we applied the Student’s t-test. Categorical variables were analyzed using the chi-square test. Additionally, the effectiveness of three distinct models was evaluated through the generation of Receiver Operating Characteristic (ROC) curves. This involved computing the Area Under the Curve (AUC), and determining the balanced sensitivity and specificity at the cut-off point that maximized the Youden index. To enhance the reliability of our findings, we calculated the 95% confidence interval (CI) for the AUC using the bootstrap method, incorporating 1000 iterations for greater accuracy. The AUC values ranged from 0.5 to 1.0. A test with an AUC of 1.0 was considered perfect. An AUC between 0.8 and 1.0 indicated a good discriminant test, while a range of 0.6 to 0.8 suggested a moderate test. An AUC from 0.5 to 0.6 was regarded as poor [22]. All statistical analyses were executed utilizing SPSS software (version 21.0), with a *p*-value threshold of 0.05 or less set for determining statistical significance.

## Results

### Patient characteristics

A total of 364 patients were initially collected from two centers. Exclusions were made for patients lacking an MRI scan (*n* = 83), missing clinical data (*n* = 19), the patient has undergone surgical treatment due to OSCC (*n* = 34), absence of blood biochemical indices (*n* = 9), and indiscernible tumor areas preventing delineation (*n* = 21) (Fig. 1). Consequently, the final training cohort comprised 160 patients (103 males and 57 females; mean age not specified), including 101 early stage and 59 late stage patients. The validation cohort consisted of 38 patients (31 males and 7 females), with 25 diagnosed at an early stage and 13 at a late stage of the condition (Table 1 and Supplementary Table S1).

**Table 1:** Characteristics of patients in training and validation cohorts

Variable	Training cohort		*p*-value	Validation cohort		*p*-value
	Early stage	Late stage		Early stage	Late stage	
Age, y (%)			0.04			0.60
≤ 63	78(77.23)	36(61.02)		20(80.00)	12(92.31)	
> 64	23(22.77)	23(38.98)		5(20.00)	1(7.69)	
Gender, n (%)			0.06			0.43
Male	71(70.30)	32(54.24)		19(76.00)	12(92.31)	
Female	30(29.70)	27(45.76)		6(24.00)	1(7.69)	
BMI			0.02			0.40
≤ 18.5	4(3.96)	10(16.95)		1(4.00)	0(0.00)	
18.5–24	65(64.36)	31(52.54)		12(48.00)	9(69.23)	
≥ 24	32(31.68)	18(30.51)		12(48.00)	4(30.77)	
PLR			0.19			0.73
≤ 150.02	65(64.36)	31(52.54)		20(80.00)	9(69.23)	
> 150.02	36(35.64)	28(47.46)		5(20.00)	4(30.77)	
NLR			0.18			0.60
≤ 3.16	84(83.17)	43(72.88)		20(80.00)	12(92.31)	
> 3.16	17(16.83)	16(27.12)		5(20.00)	1(7.69)	
LMR			0.15			0.49
≤ 5.81	59(58.42)	42(71.19)		15(60.00)	10(76.92)	
> 5.81	42(41.58)	17(28.81)		10(40.00)	3(23.08)	
SIRI			0.08			1.00
≤ 0.98	73(72.28)	34(57.63)		19(76.00)	10(76.92)	
> 0.98	28(27.72)	25(42.37)		6(24.00)	3(23.08)	

PLR: platelet-lymphocyte ratio; NLR: neutrophils-lymphocytes ratio; LMR: lymphocytes-monocytes ratio; SIRI: systemic inflammation response index; BMI: body mass index

### Univariate analysis and multivariate analysis

This study incorporated a total of 45 indicators (as shown in Supplementary Figure S1 and Supplementary Table S2). Univariate analysis revealed significant associations between several indicators and the early and late stages in the training cohort.

**Fig. 4:**
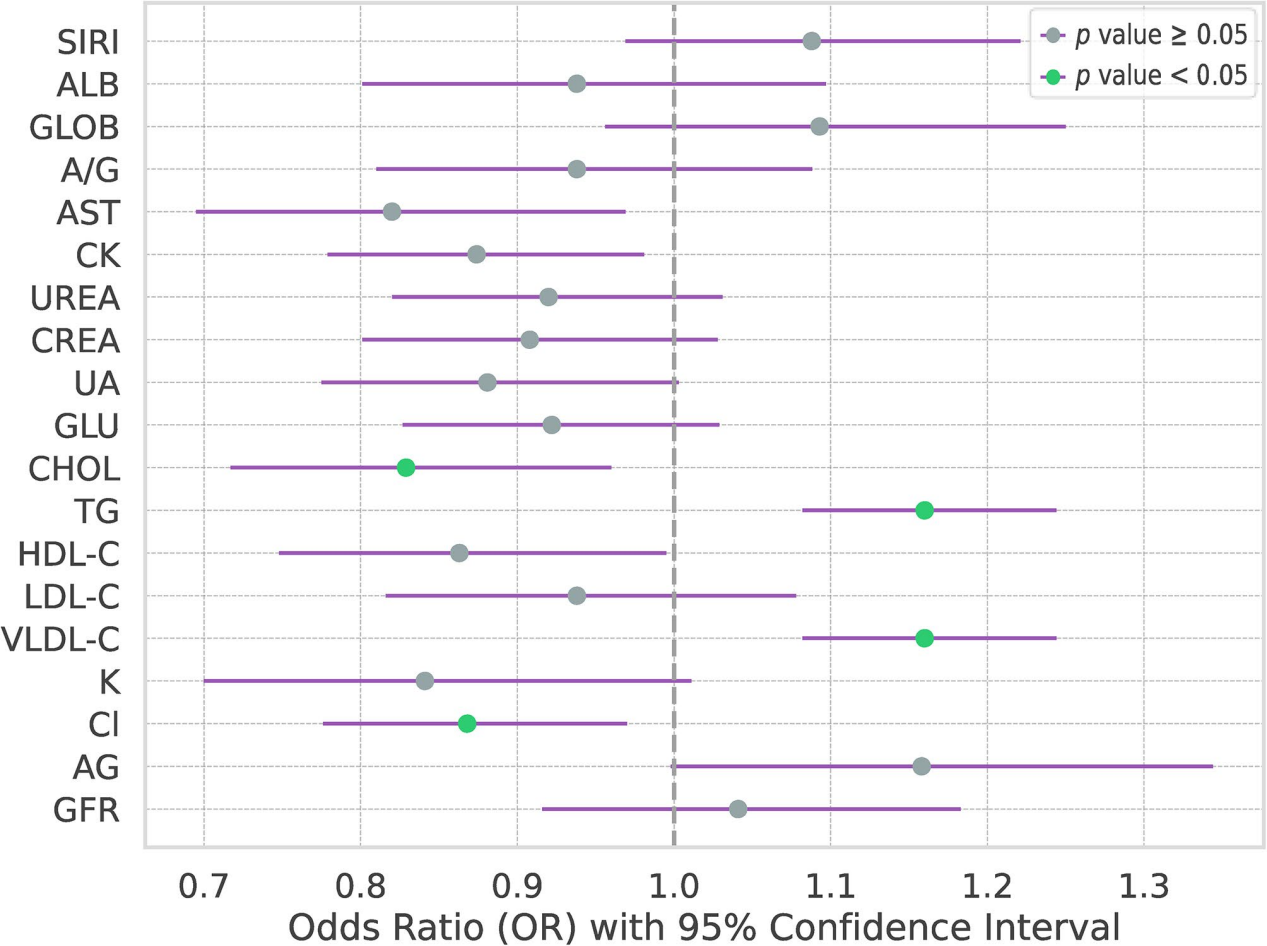
Multivariate analysis. SIRI: systemic inflammation response index; ALB: albumin; GLOB: globulin; A/G: white bulb ratio; AST: aspartate aminotransferase; CK: creatine kinase; CREA: creatinine; UA: uric acid; GLU: glucose; CHOL: cholesterol; TG: triglyceride; HDL-C: high density lipoprotein cholesterol; LDL-C: low density lipoprotein cholesterol; VLDL-C: very low density lipoprotein cholesterol; K: potassium; Cl: chlorine; AG: anion gap; GFR: glomerular filtration rate

Further, the multivariate analysis identified CHOL (OR, 0.83 [95% CI:0.72, 0.96]; *p*-value = 0.04), TG (OR, 1.16 [95% CI:1.08, 1.28]; *p*-value < 0.01), VLDL-C (OR, 1.16 [95% CI:1.08, 1.24]; *p*-value < 0.01) and Cl (OR, 0.87 [95% CI:0.78, 0.97]; *p*-value = 0.04) as independent predictors for both early and late stage outcomes (Fig. 4 and Supplementary Table S3).

### Performance evaluation of prediction models constructed using radiomics and deep learning features

**Fig. 5:**
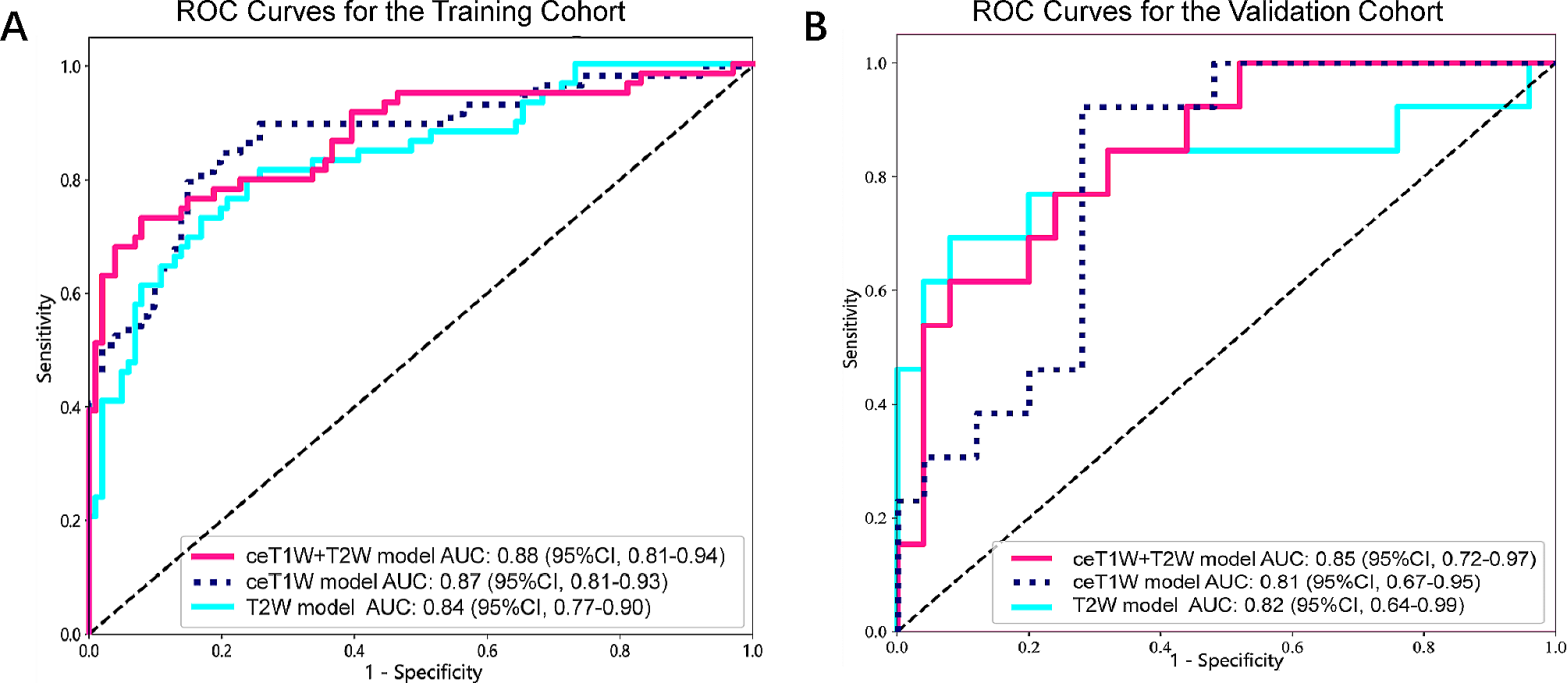
**A**: ROC curve constructed based on radiomics and deep learning features of training cohort; **B**: ROC curve constructed based on radiomics and deep learning features of validation cohort; AUC: area under ROC curve; ROC: receiver operating characteristic; CI: confidence interval; ceT1W: contrast-enhanced T1-weighted; T2W: T2-weighted

After comparing multiple deep learning models (Supplementary Table S4), we ultimately chose ViT as the final deep learning model for the construction of the fusion model. The filtered features of ceT1W model, T2W model and ceT1W + T2W model are as follows. A set of features with nonzero coefficients were selected to construct radiomics scores using a LASSO logistic regression model, as depicted in Supplementary Figure S2. The distribution of the feature scores was visualized through histograms, shown in Supplementary Figure S3 and S4. In discriminating between early and late stages, the ceT1W model demonstrated AUC values of 0.81 [95% CI: 0.67, 0.95] in the validation cohort. The T2W model recorded AUCs of 0.82 [95% CI: 0.64, 0.99]in the validation cohort. The combined ceT1W and T2W model (ceT1W + T2W) achieved AUCs of 0.85 [95% CI: 0.72, 0.97] in the validation cohort, as shown in Fig. 5. These findings demonstrate that utilizing MRI for radiomics and deep learning analysis can effectively diagnose the staging of a patient’s tumor, early or late, thereby aiding physicians in formulating surgical plans.

### Performance evaluation of prediction models constructed using radiomics, deep learning and clinical features

**Fig. 6:**
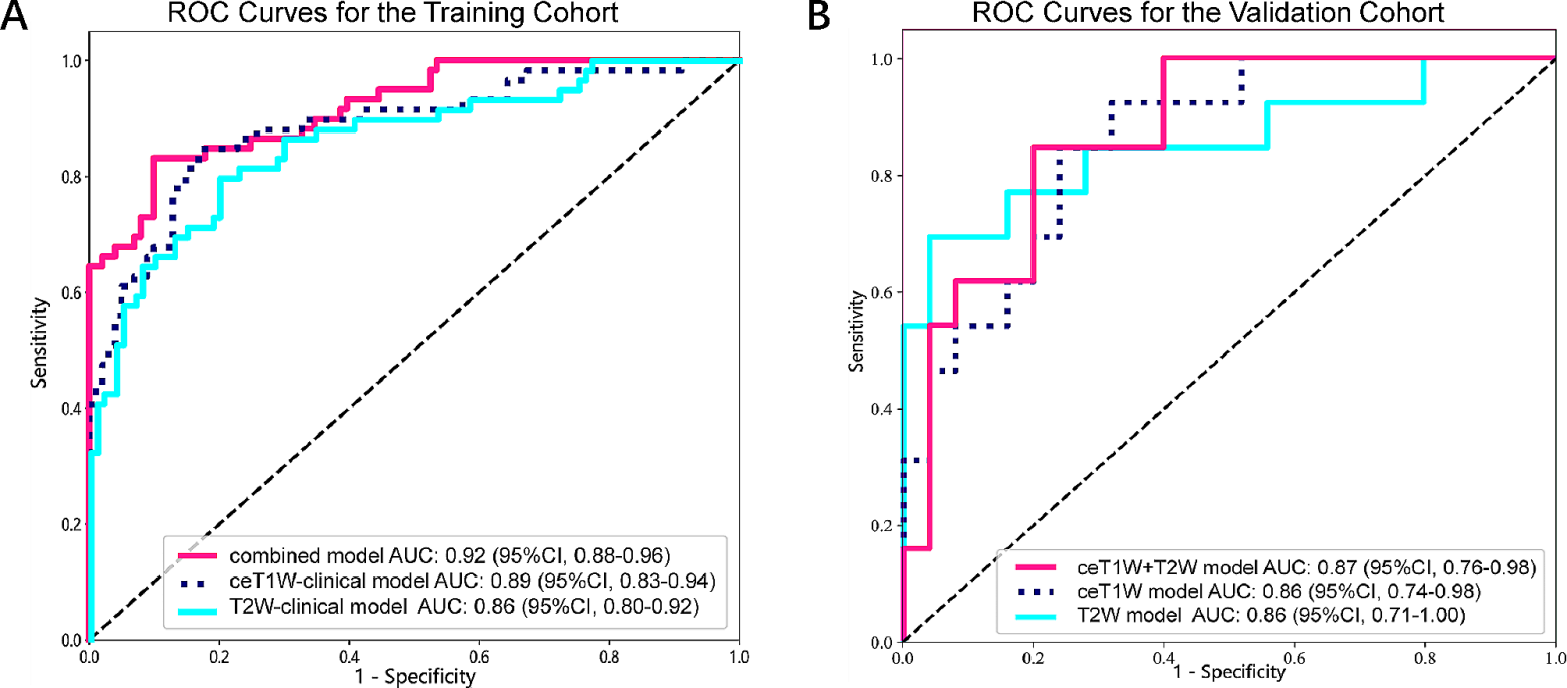
**A**: ROC curve constructed based on radiomics, deep learning and clinical features of training cohort; **B**: ROC curve constructed based on radiomics, deep learning and clinical features of validation cohort; AUC: area under ROC curve; ROC: receiver operating characteristic; CI: confidence interval; combined-mode: ceT1W + T2W-clinical model; ceT1W: contrast-enhanced T1-weighted; T2W: T2-weighted

Furthermore, the integration of clinical variables with radiomics and deep learning features resulted in an enhanced predictive performance of the combined model. Specifically, the ceT1W-clinical model demonstrated AUC scores of 0.86 [95% CI: 0.74, 0.98] in the validation cohort. Meanwhile, the T2W-clinical model recorded AUCs of 0.86 [95% CI: 0.71, 1.00] in the validation cohort. Notably, the combined model (ceT1W + T2W-clinical model) achieved AUCs of 0.87 [95% CI: 0.76, 0.98] in the validation cohort, as depicted in Fig. 6. When we incorporated extracted clinical and biochemical indicators into the model construction, the diagnostic efficacy of the model improved, with AUCs in the validation cohort increasing from 0.85 to 0.87.

## Discussion

OSCC is an aggressive malignant tumor. Due to its rapid progression, there is a significant difference in treatment protocols between early and late stage patients [23]. Furthermore, early determination of the tumor stage is crucial for patient prognosis and survival rates [24]. Therefore, identifying the stage of a patient’s tumor upon admission is vital for physicians in choosing appropriate treatment strategies. Our study offers a novel diagnostic approach based on radiomics and the ViT model, enabling rapid diagnosis of early or late stages of OSCC. Through univariate and multivariate analyses, we identified four biochemical indicators highly associated with OSCC. Finally, by integrating these extracted features into the model, we improved the diagnostic AUC, providing valuable assistance to physicians.

Our study reveals that combining biochemical analysis of lipid metabolites with radiomics and deep learning features in OSCC patients enhances the efficacy in predicting tumor stages, both early and late, compared to relying solely on radiomics and deep learning. Biochemical indicators have been proven to be of significant importance in various cancer studies. They can reflect the metabolic status, inflammatory response, and overall physiological status of tumors [25]. This study identified four markers associated with early and late diagnosis of OSCC patients through univariate and multivariate analysis, of which three were associated with lipid metabolism. This finding underscores the pivotal role of lipid metabolites in discerning OSCC progression stages. There is substantial evidence indicating that obesity is a risk factor for various cancers [26]. Research by Halczy-Kowalik and colleagues [27] found a correlation between lipid metabolism and the tumor microenvironment and grading of OSCC. Studies by Dickinson et al. [28] also pointed to elevated cholesterol levels in OSCC tissues, suggesting a disruption in the typically tightly regulated cholesterol homeostasis. Furthermore, having a BMI in the obese range is an independent risk factor for T1/2N0M0 OSCC and is associated with prognosis [27, 29]. Takasu et al. [30] pointed out that lipoprotein lipase (LPL) may regulate triglyceride levels from blood to tissue, and a decrease in LPL activity can lead to hypertriglyceridemia. And hypertriglyceridemia is associated with the risk of colorectal adenoma and colorectal cancer. The multivariate analysis revealed that serum TG levels (OR, 1.16 [95% CI:1.08, 1.28]) are associated with an increased likelihood of advanced tumor stages in patients. Emerging research suggests that hyperlipidemia, particularly elevated TG and VLDL-C levels, might contribute to a chronic inflammatory state [31], a well-documented risk factor for diverse cancers [32]. Lu et al. found that VLDL promotes breast cancer cell aggregation through enhanced migration/invasion, angiogenic activity, and anchorage-independent growth, providing a survival advantage in these conditions and promoting lung metastasis in vivo [33]. While the current evidence is insufficient to categorize TG and VLDL-C as an autonomous risk factors for OSCC, they appear to synergize with established risk factors. The multivariate analysis delineates that elevated serum VLDL-C levels (OR, 1.16 [95% CI:1.08, 1.24]) are associated with an increased likelihood of advanced tumor stages in patients, underscoring the potential role of VLDL-C in OSCC progression.

The pathogenesis of OSCC, is intricately connected to metabolic pathways. OSCC cells exhibit significant alterations in lipid metabolism. These cells augment fatty acid synthesis, which is crucial for the construction of new cellular membranes [34], energy storage [27], and signaling mechanisms [35] essential for rapid cellular growth. The multivariate analysis revealed that serum CHOL levels (OR, 0.83 [95% CI:0.72, 0.96]) are inversely associated with tumor progression in OSCC. Elevated levels of CHOL in the cell membrane have been observed in OSCC and various other tumors [36, 37]. Cholesterol-lowering drugs could play a role in inhibiting OSCC progression through multiple mechanisms [38]. This suggests that higher serum CHOL levels correlate with a decreased risk of late stage tumors. Furthermore, existing studies indicate that serum CHOL levels in OSCC patients are lower than those in healthy controls [39, 40]. Recent research by Kei et al. [41] has highlighted the complex biological interplay between blood chloride ion levels and tumor development. Their findings specifically point to the role of Cl channel dysfunction in facilitating epithelial-mesenchymal transition in OSCC. However, the exact nature of the interaction between chloride ions and OSCC remains elusive, underscoring the need for further investigation in this area. Our research findings reveal that serum CHOL levels and Cl exhibit a negative correlation with the stage of the patient’s tumor, whereas TG and VLDL-C levels are positively correlated with the tumor stage.

Ren and colleagues [42] conducted research solely using radiomics for predicting the early and late stages of head and neck squamous cell carcinoma. Zheng et al. [43] have tackled the classification issue of histological differentiation grades in patients with head and neck squamous cell carcinoma. They employed a combination of deep learning and radiomics to construct a model that demonstrates commendable predictive capabilities. This study lays a foundational groundwork for the application of radiomics and deep learning in predicting the staging and differentiation of head and neck tumor. Our study demonstrates significant predictive performance, with the radiomics and ViT models achieving an AUC of 0.88 [95% CI: 0.81, 0.94] in the training cohort and 0.85 [95% CI: 0.72, 0.97] in the validation cohort.

The ViT, as a relatively novel deep learning model, captures global image information through a self-attention mechanism, significantly enhancing its understanding of contextual relationships compared to traditional Convolutional Neural Networks (CNNs) [44]. Demonstrating strong generalization capabilities, ViT is suitable for a wide range of image tasks, including classification, object detection, and segmentation, showcasing its versatility across different scenarios [45]. While ViTs have outperformed traditional CNNs in such tasks, their application in medical image classification is still relatively novel [46]. In our research results, it was also shown that the ViT model has better predictive performance than other deep learning models. Compared to CNNs that require the design of complex convolutional kernels and pooling layers, ViT learns features directly from images through self attention mechanisms, reducing prior assumptions about image structure and being able to simultaneously process image features of different scales [47, 48]. However, the implementation of ViT models in clinical practice faces challenges such as large data requirements, high computational resources, and insufficient interpretability.

Moreover, the research delves into the exploration of multimodal methods, integrating biochemical indicators as clinical features. This comprehensive approach has yielded a combined model that outperforms the individual radiomics and deep learning models. The performance of this combined model is evidenced by its AUC of 0.92 [95% CI: 0.88, 0.96] in the training cohort and 0.87 [95% CI: 0.76, 0.98] in the validation cohort. Combining radiomics and deep learning with biochemical indicators to construct a model for predicting the staging of OSCC patients is currently an unexplored approach. This integration enhances the understanding of the pathophysiology of OSCC by identifying key biochemical indicators related to tumor progression and staging, thereby aiding in early diagnosis. By analyzing individual biochemical indicators, personalized treatment plans can be developed to tailor treatment strategies. Dynamic monitoring of these indicators during the treatment process can evaluate the treatment effect in real time and adjust it accordingly, ultimately improving patient management and achieving better treatment outcomes [49].

The research, while presenting promising avenues in the diagnosis of OSCC, has several limitations that warrant consideration. Firstly, the data utilized in our study was retrospectively collected from a multicenter cohort. Despite the multi-institutional nature of the data, the retrospective design and relatively small sample sizes of the training and validation cohorts may introduce inherent biases and hidden confounding factors. Additionally, the current scope of our model is confined to distinguishing between the early and late stages of OSCC. It does not extend to predicting patient prognosis or survival outcomes. In the future, we should include large-scale omics studies to discover more tumor related biomarkers. And improve the interpretability of the model, develop models that are easy to understand and interpret, and ensure their application in clinical practice. Solving these problems also faces many challenges. For example, obtaining sufficient and diverse data requires collaborating with multiple medical centers and coordinating data standards among different institutions. To ensure data diversity, radiomics data, biochemical indicators, genomic data, etc., should be included in the multimodal model. Additionally, technicians need to continuously optimize and adjust model parameters to improve the accuracy and stability of the model when dealing with large and complex data. If the research model reaches a high AUC after extensive testing, it can be used in clinical practice to infer disease status based on MRI and blood biochemical indicators of patients upon admission, helping physicians develop surgical and treatment strategies. In summary, our study has successfully developed and validated a preoperative lipid metabolite analysis with MRI-based model for the non-invasive prediction of tumor stages in OSCC patients. This model represents a significant step forward in the field of radiological image analysis, suggesting that transformer-based models, such as the ViT, could be a viable and promising alternative to traditional CNNs.

## Conclusions

In conclusion, our study has successfully developed and validated a novel preoperative MRI-based model for accurately predicting the stages of tumors in OSCC patients. A notable aspect of our research is the application of transformer-based models in radiomics analysis. Our findings particularly emphasize the role of lipid metabolism in OSCC progression evaluation. By enhancing the accuracy and reducing the invasiveness of OSCC stages diagnosis, our model has the potential to significantly advance the field of precision medicine.

### Electronic supplementary material

Below is the link to the electronic supplementary material.

Supplementary Material 1

## Data Availability

No datasets were generated or analysed during the current study.

## Abbreviations

OSCC: Oral Squamous Cell Carcinoma
MRI: Magnetic Resonance Imaging
ViT: Vision Transformer
GANs: Generative adversarial networks
ceT1W: Contrast-enhanced T1-weighted
T2W: T2-weighted
FLAIR: Fluid attenuation inversion recovery
BMI: Body mass index
PLR: Platelet-to-Lymphocyte Ratio
NLR: Neutrophil-to-Lymphocyte Ratio
LMR: Lymphocyte-to-Monocyte Ratio
SIRI: Systemic Immune-Inflammation Index
TBIL: Total Bilirubin
DBIL: Direct Bilirubin
IBIL: Indirect Bilirubin
TP: Total Protein
ALB: Albumin
GLOB: Globulin
A/G: Albumin/Globulin Ratio
ALT: Alanine Aminotransferase
AST: Aspartate Aminotransferase
GGT: Gamma-Glutamyl Transferase
LDH: Lactate Dehydrogenase
ALP: Alkaline Phosphatase
CK: Creatine Kinase
CK-MB: Creatine Kinase MB Isoenzyme
CREA: Creatinine
URE/CREA: Urea/Creatinine Ratio
UA: Uric Acid
GLU: Glucose
CHOL: Cholesterol
TG: Triglyceride
HDL-C: High-Density Lipoprotein Cholesterol
HDL-TC: High-Density Lipoprotein/Total Cholesterol Ratio
LDL-C: Low-Density Lipoprotein Cholesterol
VLDL-C: Very Low-Density Lipoprotein Cholesterol
APOA1: Apolipoprotein A1
APOB: Apolipoprotein B
Ca: Calcium
IP: Inorganic Phosphorus
Mg: Magnesium
HCO3: Bicarbonate
K: Potassium
Na: Sodium
Cl: Chloride
AG: Anion Gap
GFR: Glomerular Filtration Rate
TR: Repetition time
TE: Echo time
FOV: Field of view
ROI: Regions of Interest
ICCs: Intraclass correlation coefficients
GLCM: Gray-Level Co-occurrence Matrix
GLDM: Gray-Level Dependence Matrix
GLRLM: Gray-Level Run Length Matrix
GLSZM: Gray-Level Size Zone Matrix
NGTDM: Neighboring Gray Tone Difference Matrix
LASSO: Least Absolute Shrinkage and Selection Operator
ROC: Receiver Operating Characteristic
AUC: Area Under the Curve
CI: Confidence Interval
OR: Odds Ratio
CNNs: Convolutional Neural Networks
LPL: lipoprotein lipase
