# Supplementary material for: Integrating lipid metabolite analysis with MRI-based transformer and radiomics for early and late stage prediction of oral squamous cell carcinoma
Source: BMC Cancer. 2024 Jul 3;24:795. doi: 10.1186/s12885-024-12533-x (PMC11221018; doi:10.1186/s12885-024-12533-x)

Supplementary Methods:

Radiomics feature extraction

The extracted features were categorized into seven distinct types:

1. First-order features that describe the intensity distribution within the ROI, such as energy, entropy, mean, and median.
2. Two-dimensional (2D) features that characterize the ROI's size and shape, including mesh surface, pixel surface, perimeter, and maximum diameter.
3. Gray-level co-occurrence matrix (GLCM) that defines the second-order joint probability function of the ROI, covering metrics like autocorrelation, joint average, and cluster prominence.
4. Gray-level size-zone matrix (GLSZM) that counts the number of connected voxels sharing the same gray level intensity.
5. Gray-Level Dependence Matrix (GLDM) that assesses the distribution of dependencies (i.e., number of connected neighbors) for each gray level within the ROI, highlighting texture complexity and patterns.
6. Gray-level run-length matrix (GLRLM) that calculates the size of homogeneous runs for each gray level, which consists of consecutive pixels with the same gray level value.
7. Neighboring gray tone difference matrix (NGTDM) that measures the difference between a gray value and the average gray value of its neighbors within a specified distance.

In addition, 14 filters were applied to the original images, including exponential, gradient, square, square root, logarithm, lbp2D, and various wavelet filters (HLH, HLL, LHL, LLL, LHH, LLH, HHL, HHH), resulting in derived images for each patient. Except for shape features, all feature categories were computed on both the original and derived images.

Deep learning feature extraction

The deep learning model was modified to interpret the prognostic signals of tumors on MRI. Due to the millions of learnable parameters, training such a model is computationally intensive and demands a large number of images. To mitigate the issue of limited data, transfer learning can be employed to enable knowledge transfer in the deep learning model. Typically, datasets in the computer vision domain are extensive and are often utilized for model training. Accordingly, various deep learning architectures (such as DenseNet121, GoogLeNet, ResNet18, ResNet34, and ViT) were trained using the ImageNet dataset. For instance, in the DenseNet-121 model, the fully connected and softmax layers were removed, and the output values of the nodes in the last layer were used as deep learning features.

Radiomics and deep learning signature building

For ceT1W model, total 1857 features were extracted from each tumor ROI, including 1024 DL features (ViT model) and 833 radiomics features, respectively. Of them, 837 and 748 features with high reproducibility and stability (ICC > 0.75), were reduced to 582 and 419 potential predictors by the Spearman's rank correlation coefficient analysis for the DL and radiomics features, respectively. After deleting features with correlation coefficients exceeding 0.9, there are 24 and 143 remaining features, respectively. After the LASSO logistic regression analysis, 2 and 15 features were finally selected to derive the DL signature and radiomics signature, respectively. The selected features for construction of radiomics and DL signature were listed in Supplementary Figure S2.

For T2W model, total 1857 features were extracted from each tumor ROI, including 1024 DL features (ViT model) and 833 radiomics features, respectively. Of them, 875 and 716 features with high reproducibility and stability (ICC > 0.75), were reduced to 527 and 301 potential predictors by

the Spearman's rank correlation coefficient analysis for the DL and radiomics features, respectively. After deleting features with correlation coefficients exceeding 0.9, there are 39 and 135 remaining features, respectively. After the LASSO logistic regression analysis, 4 and 15 features were finally selected to derive the DL signature and radiomics signature, respectively. The selected features for construction of radiomics and DL signature were listed in Supplementary Figure S2.

For ceT1W+T2W model, total 3714 features were extracted from each tumor ROI, including 2048 DL features (ViT model) and 1666 radiomics features, respectively. Of them, 1075 and 1274 features with high reproducibility and stability (ICC > 0.75), were reduced to 724 and 536 potential predictors by the Spearman's rank correlation coefficient analysis for the DL and radiomics features, respectively. After deleting features with correlation coefficients exceeding 0.9, there are 57 and 286 remaining features, respectively. After the LASSO logistic regression analysis, 2 and 9 features were finally selected to derive the DL signature and radiomics signature, respectively. The selected features for construction of radiomics and DL signature were listed in Supplementary Figure S2.

Supplementary Table S1: TNM stage of patients in training and validation cohorts.

Variable	Training cohort	Validation cohort	*p*-value
T			0.12
1	71(44.38)	23(60.53)	
2	80(50.00)	12(31.58)	
3	9(5.62)	3(7.89)	
N			0.42
0	101(63.16)	26(68.42)	
1	23(14.37)	7(18.42)	
2	36(22.50)	5 (13.16)	
TNM			0.20
1	60(37.50)	19(50.00)	
2	41(25.62)	6(15.79)	
3	23(14.37)	8(21.05)	
4	36(22.50)	5(13.16)	

All patients included in this study had an M stage of M0.

Supplementary Table S2: Univariate analysis.

Variable	OR	95%CI	*p*-value	Variable	OR	95%CI	*p*-value
Gender	1.12	0.99-1.27	0.13	CREA	0.84	0.74-0.94	0.01
Age	1.14	1.00-1.29	0.11	UREA/CREA	0.92	0.81-1.03	0.21
PLR	1.15	1.02-1.30	0.05	UA	0.85	0.74-0.97	0.05
NLR	1.15	0.99-1.34	0.11	GLU	0.86	0.77-0.97	0.04
LMR	0.87	0.77-0.98	0.06	CHOL	0.81	0.72-0.92	0.01
SIRI	1.19	1.05-1.35	0.02	TG	1.24	1.07-1.44	0.02
BMI	0.90	0.81-1.00	0.08	HDL-C	0.78	0.67-0.90	0.01
TBIL	0.90	0.79-1.01	0.14	HDL-TC	1.24	1.03-1.48	0.05
DBIL	0.94	0.84-1.06	0.40	LDL-C	0.82	0.73-0.92	0.01
IBIL	0.90	0.80-1.02	0.15	VLDL-C	1.24	1.07-1.44	0.02
TP	1.16	1.01-1.34	0.08	APOA1	0.89	0.79-1.00	0.11
ALB	0.78	0.67-0.91	0.01	APOB	0.87	0.78-0.98	0.05
GLOB	1.17	1.05-1.32	0.02	APOA1/B	1.11	0.98-1.27	0.18
A/G	0.84	0.74-0.96	0.03	Ca	1.07	0.95-1.21	0.32
ALT	1.17	0.98-1.41	0.14	IP	1.12	1.00-1.26	0.10
AST	0.78	0.65-0.93	0.02	Mg	0.92	0.80-1.05	0.29
ALT/AST	0.91	0.80-1.04	0.23	HCO3	1.14	0.99-1.31	0.13
GGT	0.95	0.84-1.07	0.45	K	0.72	0.59-0.87	0.01
LDH	1.19	1.01-1.41	0.08	Na	0.92	0.82-1.04	0.25
ALP	1.17	1.00-1.37	0.09	Cl	0.83	0.74-0.94	0.01
CK	0.79	0.70-0.89	0.01	AG	1.27	1.08-1.48	0.01
CK-MB	1.06	0.94-1.19	0.43	GFR	1.19	1.05-1.36	0.03
UREA	0.80	0.71-0.90	0.01				

OR: Odds Ratio; CI: Confidence Interval; PLR: platelet-lymphocyte ratio; NLR: neutrophils-lymphocytes ratio; LMR: lymphocytes-monocytes ratio; SIRI: systemic inflammation response index; BMI: body mass index; TBIL: total bilirubin; DBIL: direct bilirubin; IBIL: Indirect bilirubin; TP: total protein; ALB: albumin; GLOB: globulin; A/G: white bulb ratio; ALT: alanine aminotransferase; AST: aspartate aminotransferase; GGT: γ- glutamyl transpeptidase; LDH: lactate dehydrogenase; ALP: alkaline phosphatase; CK: creatine kinase; CREA: creatinine; UA: uric acid; GLU: glucose; CHOL: cholesterol; TG: triglyceride; HDL-C: high density lipoprotein cholesterol; HDL-TC: high density lipoprotein total cholesterol; LDL-C: low density lipoprotein cholesterol; VLDL-C: very low density lipoprotein cholesterol; APOA1: apolipoprotein A1; APOB: apolipoprotein B; Ca: calcium; IP: inorganic phosphate; Mg: magnesium; HCO3: bicarbonate; K: potassium; Na: sodium; Cl: chlorine; AG: anion gap; GFR: glomerular filtration rate.

Supplementary Table S3: Multivariate analysis.

Variable	OR	95%CI	*p*-value
SIRI	1.09	0.97-1.22	0.24
ALB	0.94	0.80-1.10	0.50
GLOB	1.09	0.96-1.25	0.27
A/G	0.94	0.81-1.09	0.48
AST	0.82	0.70-0.97	0.05
CK	0.87	0.78-0.98	0.06
UREA	0.92	0.82-1.03	0.23
CREA	0.91	0.80-1.03	0.20
UA	0.88	0.78-1.00	0.11
GLU	0.92	0.83-1.03	0.22
CHOL	0.83	0.72-0.96	0.04
TG	1.16	1.08-1.24	< 0.01
HDL-C	0.86	0.75-1.00	0.09
LDL-C	0.94	0.82-1.08	0.45
VLDL-C	1.16	1.08-1.24	<0.01
K	0.84	0.70-1.01	0.12
Cl	0.87	0.78-0.97	0.04
AG	1.16	1.00-1.34	0.11
GFR	1.04	0.92-1.18	0.61

OR: Odds Ratio; CI: Confidence Interval; SIRI: systemic inflammation response index; ALB: albumin; GLOB: globulin; A/G: white bulb ratio; AST: aspartate aminotransferase; CK: creatine kinase; CREA: creatinine; UA: uric acid; GLU: glucose; CHOL: cholesterol; TG: triglyceride; HDL-C: high density lipoprotein cholesterol; LDL-C: low density lipoprotein cholesterol; VLDL-C: very low density lipoprotein cholesterol; K: potassium; Cl: chlorine; AG: anion gap; GFR: glomerular filtration rate.

Supplementary Table S4: Comparison between deep learning models.

Model-ceT1W	AUC (95%CI)	Model-T2W	AUC (95%CI)
ViT	0.82 (0.67-0.96)	ViT	0.75 (0.55-0.94)
DenseNet121	0.75 (0.57-0.94)	DenseNet121	0.66 (0.47-0.84)
GoogLeNet	0.67 (0.50-0.85)	GoogLeNet	0.73 (0.57-0.89)
ResNet18	0.71 (0.53-0.90)	ResNet18	0.60 (0.42-0.78)
ResNet34	0.72 (0.55-0.89)	ResNet34	0.68 (0.50-0.86)

ViT: vision transformer; CI: confidence interval; ceT1W: contrast-enhanced T1-weighted; T2W: T2-weighted.

Supplementary Figure S1 Univariate analysis. PLR: platelet−lymphocyte ratio; NLR: neutrophils−lymphocytes ratio; LMR: lymphocytes−monocytes ratio; SIRI: systemic inflammation response index; BMI: body mass index; TBIL: total bilirubin; DBIL: direct bilirubin; IBIL: Indirect bilirubin; TP: total protein; ALB: albumin; GLOB: globulin; A/G: white bulb ratio; ALT: alanine aminotransferase; AST: aspartate aminotransferase; GGT: γ- glutamyl transpeptidase; LDH: lactate dehydrogenase; ALP: alkaline phosphatase; CK: creatine kinase; CREA: creatinine; UA: uric acid; GLU: glucose; CHOL: cholesterol; TG: triglyceride; HDL-C: high density lipoprotein cholesterol; HDL-TC: high density lipoprotein total cholesterol; LDL-C: low density lipoprotein cholesterol; VLDL-C: very low density lipoprotein cholesterol; APOA1: apolipoprotein A1; APOB: apolipoprotein B; Ca: calcium; IP: inorganic phosphate; Mg: magnesium; HCO3: bicarbonate; K: potassium; Na: sodium; Cl: chlorine; AG: anion gap; GFR: glomerular filtration rate.

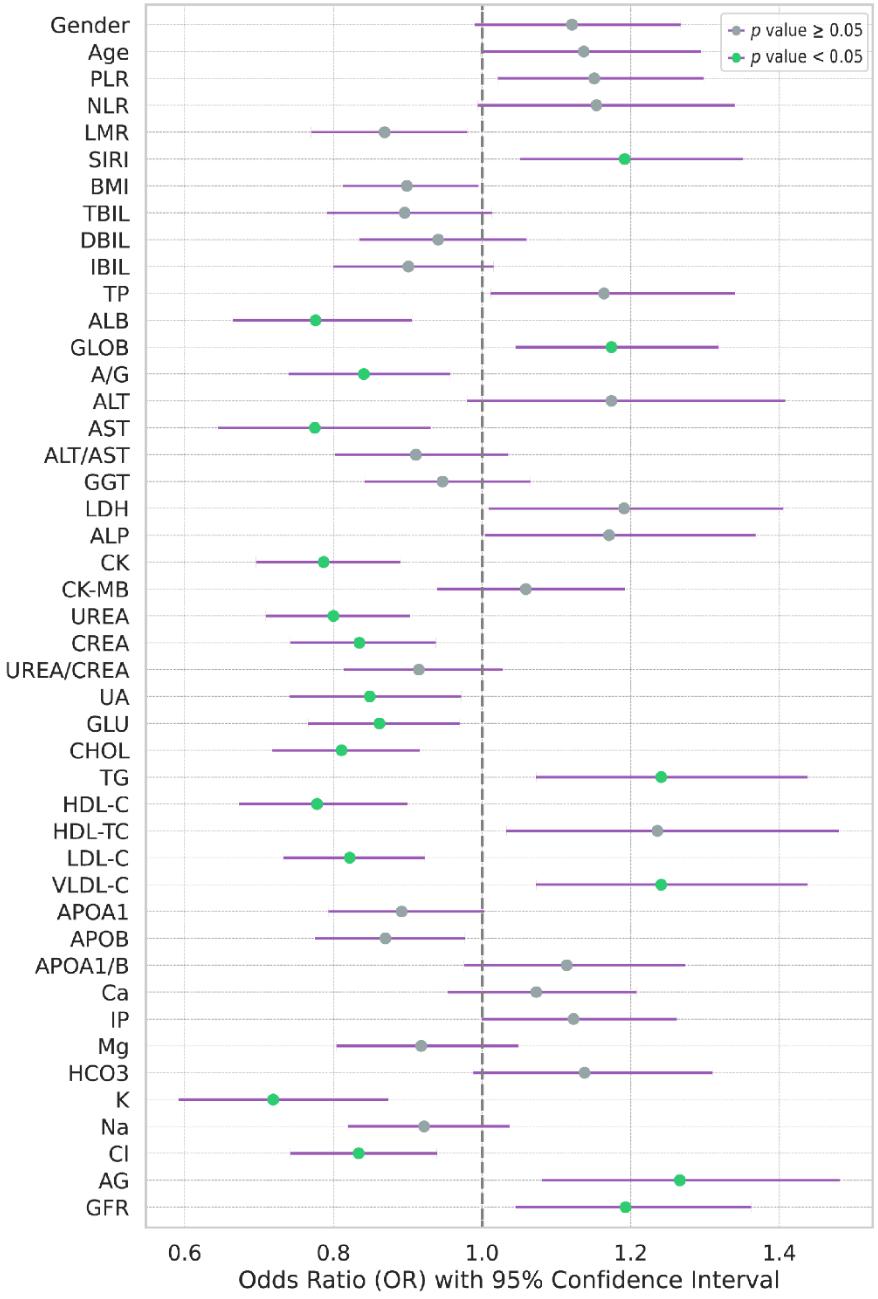

Supplementary Figure S2. Mean square error of cross-validation of LASSO model. A: ceTIW model. B: T2W model. C: ceTIW+T2W model. MSE: mean square error.

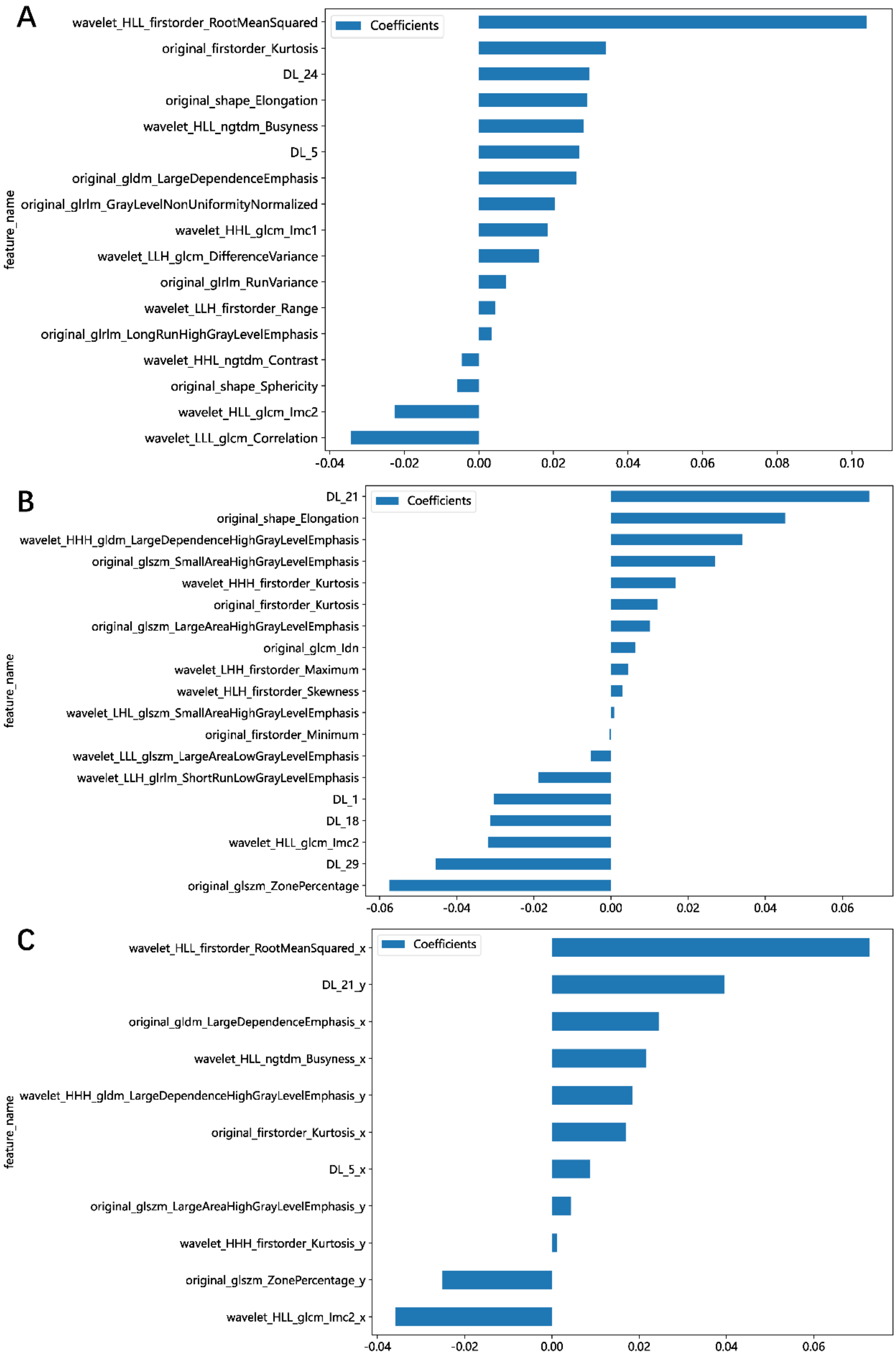

Supplementary Figure S3. LASSO coefficient solution path of features. A: ceTIW model. B: T2W model. C: ceTIW+T2W model. MSE: mean square error.

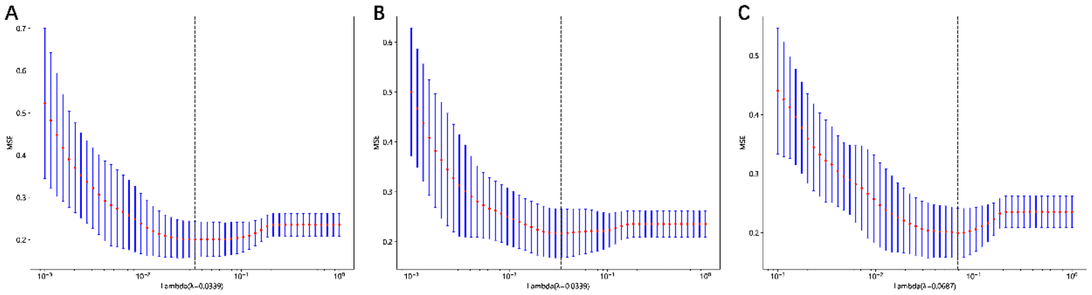

Supplementary Figure S4. The histogram of the feature score. The y-axis indicates the selected six radiomics features, and the x-axis represents the coefficients of LASSO model. A: ceTIW model. B: T2W model. C: ceTIW+T2W model. MSE: mean square error. LASSO: the least absolute shrinkage and selection operator; LLL: low-low-low-pass filtered image; LHH: low-high-high-pass filtered image; LLH: low-low-high-pass filtered image; LHL: low-high-low-pass filtered image; HHL: high-high-low-pass filtered image; HLL: high-low-low-pass filtered image; HLH: high-low- high-pass filtered image; HHH: high-high-high-pass filtered image.

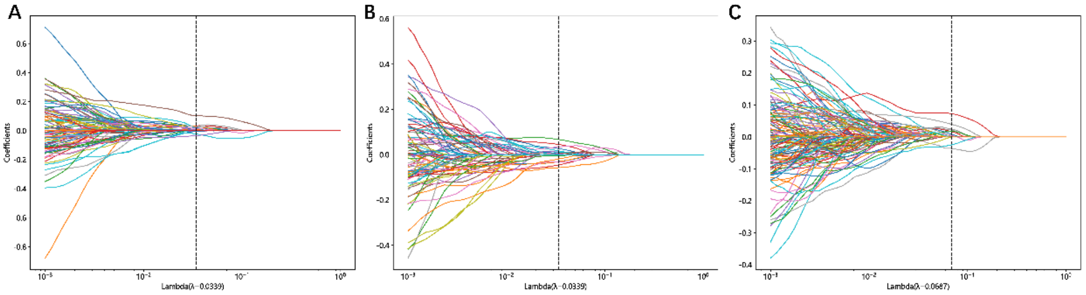

Supplement: Supplementary file 1 — Supplementary Material 1 [file 12885_2024_12533_MOESM1_ESM.pdf]
